# Type 2 Diabetes in Young Females Results in Increased Serum Amyloid A and Changes to Features of High Density Lipoproteins in Both HDL_2_ and HDL_3_

**DOI:** 10.1155/2017/1314864

**Published:** 2017-05-09

**Authors:** Kayleigh Griffiths, Agnieszka Pazderska, Mohammed Ahmed, Anne McGowan, Alexander P. Maxwell, Jane McEneny, James Gibney, Gareth J. McKay

**Affiliations:** ^1^Centre for Public Health, Queen's University Belfast, Belfast, UK; ^2^Department of Endocrinology, Tallaght Hospital, Dublin 24, Ireland

## Abstract

Persons with type 2 diabetes mellitus (T2DM) have an elevated risk of atherosclerosis. High-density lipoproteins (HDL) normally protect against cardiovascular disease (CVD), but this may be attenuated by serum amyloid A (SAA). In a case-control study of young females, blood samples were compared between subjects with T2DM (*n* = 42) and individuals without T2DM (*n* = 42). SAA and apolipoprotein AI (apoAI) concentrations, paraoxonase-1 (PON-1), cholesteryl ester transfer protein (CETP), and lecithin-cholesterol acyltransferase (LCAT) activities were measured in the serum and/or HDL_2_ and HDL_3_ subfractions. SAA concentrations were higher in T2DM compared to controls: serum (30 mg/L (17, 68) versus 15 mg/L (7, 36); *p* = 0.002), HDL_2_ (1.0 mg/L (0.6, 2.2) versus 0.4 mg/L (0.2, 0.7); *p* < 0.001), and HDL_3_, (13 mg/L (8, 29) versus 6 mg/L (3, 13); *p* < 0.001). Serum-PON-1 activity was lower in T2DM compared to that in controls (38,245 U/L (7025) versus 41,109 U/L (5690); *p* = 0.043). CETP activity was higher in T2DM versus controls in HDL_2_ (232.6 *μ*mol/L (14.1) versus 217.1 *μ*mol/L (25.1); *p* = 0.001) and HDL_3_ (279.5 *μ*mol/L (17.7) versus 245.2 *μ*mol/L (41.2); *p* < 0.001). These results suggest that individuals with T2DM have increased SAA-related inflammation and dysfunctional HDL features. SAA may prove to be a useful biomarker in T2DM given its association with elevated CVD risk.

## 1. Introduction

The prevalence of type 2 diabetes mellitus (T2DM) in younger adults is increasing, such that in the UK between 1991 and 2010, the prevalence of T2DM rose from 5.9% to 12.4% in persons < 40 years of age [1]. Subjects with T2DM are at increased risk of developing cardiovascular disease (CVD) [2]. Furthermore, those with early-onset T2DM, commonly aged < 45 years have an even greater risk of developing macrovascular complications [3]. There are sustained efforts to identify biomarkers that can be used to direct targeted management to prevent or delay the onset of diabetes and/or the development of CVD [4]. Lipoprotein levels are partly regulated by insulin, and altered lipoprotein profiles in those with T2DM are associated with increased inflammation and oxidative stress and implicated in the subsequent development of atherosclerotic and diabetic complications [5]. Lower high-density lipoprotein-cholesterol (HDL-C) levels are associated with CVD, and decreased HDL-C levels are frequently reported in subjects with T2DM; however, increasing circulating HDL-C has not directly reduced the rate of CVD events, suggesting the functional aspects of HDL may be more important than the absolute circulating HDL-C level in the development of CVD [6, 7].

Under normal circumstances, HDL is proposed to be antiatherogenic, displaying anti-inflammatory and antioxidant properties through its utilisation of several associated proteins and enzymes. These include apolipoprotein AI (apoAI) that is essential for reverse cholesterol transport (RCT) and paraoxonase-1 (PON-1), which has the ability to prevent oxidative damage of both HDL itself and low-density lipoprotein (LDL) [8]. Also, cholesteryl ester transfer protein (CETP) acts to promote the exchange of triglycerides (TGs) and cholesterol esters (CEs) between HDL particles and LDL/very low density lipoprotein (VLDL), resulting in the removal of CEs from the circulation for subsequent excretion [9]. Finally, lecithin-cholesterol acyltransferase (LCAT) is responsible for the esterification of free cholesterol and thus the maturation of HDL with some evidence to support a role in the antioxidative activity of HDL [10, 11]. These associated proteins confer HDL with very heterogeneous physiochemical and functional properties, and changes in the ratio of these proteins will affect the various antiatherogenic functions of HDL [12].

HDL has been reported to become dysfunctional under inflammatory conditions, and the release of serum amyloid A (SAA), an inflammatory marker whose concentration can increase 1000-fold in response to injury or inflammation, associates with HDL, in particular, the smaller HDL_3_ subfraction [13]. The association of HDL with SAA leads to a change in its protein and lipid composition, and potentially causes the HDL molecule to become dysfunctional, losing several protective properties, thus promoting the development of CVD [11, 14–16].

T2DM is associated with increased inflammation, and studies have reported increased serum-SAA in subjects with T2DM, compared to that in healthy controls [17, 18]. As such, we sought to evaluate the utility of SAA as a biomarker of inflammation in young females with T2DM, given the limited literature available and the increased incidence of T2DM earlier in life with higher associated risk of diabetic and cardiovascular complications. In addition, given HDL-SAA is a more stable marker of chronic inflammation than serum-SAA alone [19], and as CVD correlates with HDL subfraction dysfunction [14, 20], we chose to directly measure functional components of HDL in subjects with T2DM, namely, the activities of HDL-PON-1, CETP, and LCAT.

## 2. Methods and Materials

Favourable ethical opinion was obtained from the Research Ethics Committee of Adelaide and Meath Hospital and St James' Hospital, Dublin, Ireland. Written informed consent was obtained from each participant included in the study, and the study protocol conformed to the ethical guidelines of the 1975 Declaration of Helsinki.

### 2.1. Patient Population

A total of 84 subjects, without clinical evidence of CVD, were recruited. All participants were female, either with T2DM (*n* = 42) or controls without T2DM (*n* = 42), matched for age and body mass index (BMI). Participants with T2DM were prescribed metformin (26), sulfonylurea (7), dipeptidyl peptidase-4 (DDP4) inhibitors (4), glucagon-like peptide-1 (GLP-1) analogue (8), and insulin (5), with 13 of the T2DM subjects being managed with diet alone.

### 2.2. Exclusion Criteria

Participants using statin medications were excluded to minimise possible confounding effects on lipid metabolism. All participants recruited had no known CVD.

### 2.3. Blood Processing

Peripheral blood was obtained from fasting subjects by venepuncture using a vacuette system. Serum was isolated following centrifugation (Beckman J-6B centrifuge) at 3000 rpm for 15 minutes at 4°C. The resulting supernatant was removed and immediately frozen in 1.3 mL aliquots at −80°C, until required.

### 2.4. Primary Subject Analysis

Baseline measurements included fasting serum levels of glucose, insulin, total cholesterol, triglycerides, HDL cholesterol, and LDL cholesterol, which were measured using standard enzymatic assays on an automated ILab-600 biochemical analyser (Cobas Roche Diagnostics, West Sussex, UK). HbA1c was measured in serum by ion exchange HPLC. Height (cm) and weight (kg) were collected using a stadiometer and calibrated scales and used to determine BMI (kg/m^2^).

### 2.5. Isolation of LDL/VLDL, HDL_2_, and HDL_3_ from Serum

LDL/VLDL, HDL_2_, and HDL_3_ were isolated from freshly thawed serum by a rapid ultracentrifugation procedure at 100,000 rpm according to the method of McPherson et al. [21]. This was a 3-step procedure, taking 6 hours in total. Firstly, mixed LDL/VLDL and crude HDL were isolated from serum by rapid flotation and sedimentation, followed by isolation of HDL_2_ and HDL_3_ via two rapid flotation steps. Lipoproteins were stored immediately at −80°C until required for analysis.

### 2.6. ApoAI Determination

ApoAI concentration in HDL_2_ and HDL_3_ was determined using single radial immunodiffusion (SRID) as described by McPherson et al. [21]. The coefficients of variation (CVs) for apoAI were 3.2% and 5.4% (interassay) and 7.8% and 4.0% (intra-assay) for HDL_2_ and HDL_3_, respectively.

### 2.7. Serum, HDL_2_, and HDL_3_-SAA

The commercially available ELISA procedure (Human SAA KHA0011C, Invitrogen, California, USA) was used to measure SAA concentration in serum, HDL_2_, and HDL_3_, specifically detecting SAA1. As per the manufacturer's instructions, analysis was performed on a TRITURUS ELISA system (Grifols, Vicopisano, Italy). Prior to analyses, samples were diluted as follows: serum, 1 : 150; HDL_2_, 1 : 10; and HDL_3_, 1 : 100. The CVs for SAA were 2.8%, 3.6%, and 5.6% (interspecific) and 8.0%, 9.5%, and 11% (intraspecific) for SAA, HDL_2_, and HDL_3_, respectively.

### 2.8. Conjugated Diene Concentration

The concentration of conjugated dienes (CDs) was evaluated by measuring the absorbance at 234 nm using a spectrophotometric plate reader (SpectraMax 190, Molecular Devices Inc.). Briefly, samples were diluted 1/10 using a sodium chloride solution (density 1.006 g/mL) and 300 *μ*L was pipetted into a U/V 96-well plate (Costar, Corning, NY, USA). The Beer-Lambert Law was used to calculate concentration:

(1) $$\begin{array}{c} A=\varepsilon CL, \end{array}$$

where *A* is the absorbance (nm), *ε* is the extinction coefficient (2.95 × 10^4^ M^−1^ cm^−1^), *C* is the concentration (M), and *L* is the path length (1 cm). The CVs for CDs were 11.8%, 5.8%, and 8.3% (interassay) and 2.4%, 1.0%, and 0.6% (intra-assay) for LDL/VLDL, HDL_2_, and HDL_3_, respectively.

### 2.9. PON-1 Activity

This method was an adaptation from the methodology according to Hasselwander and colleagues [22]. Paraoxonase arylesterase activity was determined via its ability to hydrolyse phenyl acetate. Serum (5 *μ*L), HDL_2_ (200 *μ*L), and HDL_3_ (20 *μ*L) were adjusted to 2700 *μ*L with assay buffer (20 mM Tris-HCl, pH 8.0 containing 1.0 mM CaCl_2_), followed by the addition of 300 *μ*L of 10 mM phenyl acetate. 300 *μ*L of this solution was then dispensed in duplicate into a 96-well UV plate. The production of phenol was monitored spectrophotometrically at 25°C at *λ* = 270 nm in a plate reader for 5 minutes at 10-second intervals. Change in absorbance was noted after 3 minutes. The following equation was used to determine arylesterase activity (U/L):

(2) $$\begin{array}{c} arylesterase\;activity\;\left(U/L\right)=\frac{\Delta A\times V_{t}}{T\times \varepsilon \times V_{s}}\times 10^{3}, \end{array}$$

where ∆*A* is the change in absorbance, *V*_t_ is the total volume of the sample in mL (3.0 mL), *T* is the time in minutes, *ε* is the extinction coefficient (1310 M^−1^ cm^−1^), and *V*_s_ is the sample volume in mL (0.005 mL for serum, 0.2 mL for HDL_2_, and 0.02 mL for HDL_3_). One unit of arylesterase activity (U) was defined as 1 *μ*mol of phenol generated per minute. The CVs for PON-1 were 3.8%, 8.0%, and 4.2% (interassay) and 6.4%, 3.1%, and 2.3% (intra-assay) for serum, HDL_2_, and HDL_3_, respectively.

### 2.10. CETP and LCAT Activity

CETP and LCAT activities were analysed in HDL_2_ and HDL_3_ using commercially available fluorometric assays, as per the manufacturer's instructions (CETP catalogue number RB-CETP and LCAT catalogue number RB-LCAT, ROAR Biomedical, NY, USA). A FLUOstar OPTIMA plate reader (BMG Labtech, Germany) (*E*_x_ = 465 nm; *E*_m_ = 535 nm) was used to measure the rate of CE transfer from the synthetic donor particles to the acceptor molecule. The fluorescence intensity was then determined by subtracting the blank fluorescence intensity from each sample and read against the standard curve. Units are expressed in *μ*moles/L for absolute values. LCAT results were reported as a ratio of the two emission intensities (470/390 nm), where the nonhydrolysed substrate excitation was read at *E*_m_ = 470 nm and the hydrolysed substrate emission fluorescence was read at *E*_m_ = 390 nm using a FLUOstar OPTIMA plate reader. The CVs for CETP were 3.7% and 6.2% (interassay) and 1.6% and 1.0% (intra-assay), for HDL_2_ and HDL_3_, respectively. The CVs for LCAT were 4.4% and 0.7% (interassay) and 0.9% and 0.9% (intra-assay) for HDL_2_ and HDL_3_, respectively.

### 2.11. Statistical Analysis

Statistical analyses were performed using SPSS version 21 (IBM SPSS Statistics for Windows, Version 21.0. Armonk, NY: IBM Corp.). Individual variables were assessed for normality of distribution and, if required, were logarithmically transformed. Results were expressed as mean (standard deviation (SD)) if normally distributed or geometric mean (interquartile range (IQ)) if logarithmic transformation. An independent *t*-test was used to determine between group differences and correlations were performed using Pearson's coefficient. Nonstandardised results are presented, where concentrations of HDL_2_- and HDL_3_-associated proteins were calculated per volume of serum, with apoAI-standardised results appearing in Appendix 1 in Supplementary Material available online at https://doi.org/10.1155/2017/1314864.

## 3. Results

### 3.1. Subject Characteristics

Subject characteristics are shown in Table 1. There were no significant differences between T2DM and control subjects with respect to mean age, BMI, total cholesterol, triglycerides, and LDL-cholesterol, *p* > 0.05 for all comparisons. However, T2DM subjects had higher levels of fasting glucose, 7.78 mmol/L (6.10, 9.35) versus 4.94 mmol/L (4.60, 5.13); *p* < 0.001, and HbA1c, 7.26% (6.10, 8.05) versus 5.42% (5.13, 5.60); *p* < 0.001, and lower HDL-C, 1.03 mmol/L (0.89, 1.14) versus 1.20 mmol/L (1.08, 1.34); *p* = 0.004, compared to the control subjects.

### 3.2. ApoAI Concentration in HDL_2_ and HDL_3_

No significant difference was found between T2DM subjects and control subjects with regard to apoAI concentration in either HDL_2_ or HDL_3_, *p* > 0.05 for both comparisons (Table 2).

### 3.3. SAA in Serum, HDL_2_, and HDL_3_

SAA concentration was significantly higher in subjects with T2DM, compared to controls in serum (30 mg/L (17, 68) versus 15 mg/L (7, 36); *p* = 0.002), HDL_2_ (1.0 mg/L (0.6, 2.2) versus 0.4 mg/L (0.2, 0.7); *p* < 0.001), and HDL_3_ (13 mg/L (8, 29) versus 6 mg/L (3, 13); *p* < 0.001) (Table 2).

### 3.4. PON-1 in HDL_2_ and HDL_3_

PON-1 activity in serum was lower in subjects with T2DM, compared to that in control subjects (38,245 U/L (7025) (41,109 U/L (5690)), *p* = 0.043). However, when subjects taking insulin or GLP-1 analogues were excluded, serum-PON-1 activity was not significantly different between the groups (*p* > 0.05). In HDL_2_ and HDL_3_, there was no difference in the activity of PON-1 (*p* > 0.05 for both comparisons) (Table 2).

A subanalysis excluding those taking insulin and/or GLP-1 analogues found no significant change in any reported associations except between group analyses of serum-SAA and serum-PON-1 activity, which was no longer significant.

### 3.5. CETP Activity in HDL_2_ and HDL_3_

CETP activity in HDL_2_ was higher in subjects with T2DM compared to that in control subjects (232.6 *μ*mol/L (14.1) 217.1 *μ*mol/L (25.1), *p* = 0.001). This was also the case in HDL_3_, with CETP activity being higher in the T2DM subjects compared to that in the controls (279.5 *μ*mol/L (17.7), 245.2 *μ*mol/L (41.2), *p* < 0.001) (Table 2).

### 3.6. LCAT Activity in HDL_2_ and HDL_3_

No significant difference in LCAT activity was found between the T2DM subjects and the control subjects in either HDL_2_ or HDL_3_, *p* > 0.05, for both comparisons (Table 2).

### 3.7. Correlations between SAA and Subject Characteristics

Correlations compared data from all 84 subjects (Table 3). BMI showed a positive correlation with SAA concentration in serum (*r* = 0.480; *p* = 0.001), HDL_2_ (*r* = 0.358; *p* = 0.001), and HDL_3_ (*r* = 0.414; *p* < 0.001). Fasting glucose was positively correlated with SAA concentration in serum (*r* = 0.294; *p* = 0.009) and HDL_3_ (*r* = 0.265; *p* = 0.021). HbA1c was positively correlated with SAA concentration in serum (*r* = 0.348; *p* = 0.002) and HDL_3_ (*r* = 0.302; *p* = 0.008). Age and HDL-C did not show any correlation with SAA concentration (*p* < 0.05). There was a strong positive correlation between serum-SAA and HDL_2_-SAA (*r* = 0.682, *p* < 0.001) and a much stronger relationship between serum-SAA and HDL_3_-SAA subfraction (*r* = 0.906, *p* < 0.001).

Correlations between SAA- and HDL-associated enzymes are presented in Table 4. No significant correlation was detected between serum-SAA and serum-PON-1 activity (*p* > 0.05). The concentration of HDL_2_-SAA was not related to the activity of HDL_2_-PON-1, HDL_2_-CETP, or HDL_2_-LCAT (*p* > 0.05). The concentration of HDL_3_-SAA was also not related to the activity of HDL_3_-PON-1 or HDL_3_-LCAT (*p* > 0.05). However, results illustrated a positive correlation between HDL_3_-SAA association and HDL_3_-CETP activity (*r* = 0.333; *p* = 0.002). Given HbA1c was significantly associated with serum-SAA, correlations between HbA1c- and HDL-associated proteins and enzymes were considered (Table 5). Only CETP activity was significantly correlated with HbA1c in both HDL_2_ (*r* = 0.269, *p* = 0.018) and HDL_3_ (*r* = 0.296, *p* = 0.009). Positive associations between both LDL/VLDL-CD and serum-SAA and LDL/VLDL-CD and HDL_3_-SAA were detected (*r* = 0.302, *p* = 0.006 and *r* = 0.245, *p* = 0.028, resp.), although there was no significant difference in CD concentration for LDL/VLDL, HDL_2_, or HDL_3_ (*p* > 0.05) between T2DM and controls (data not shown).

## 4. Discussion

Subjects with T2DM have increased inflammation and an enhanced risk of developing atherosclerosis and subsequent CVD, with those who develop T2DM before 45 years of age being at greater risk of macrovascular complications than those developing T2DM after 45 years of age [3]. Overall, our results demonstrate increased SAA-related inflammation and abnormalities in HDL-associated enzymes in young female subjects with T2DM.

Our study focused solely on young premenopausal females with T2DM. Normally, premenopausal women benefit from the protective properties of oestrogen against developing CVD [23]. However, our study suggests that variation in HDL features may contribute to the loss of the cardioprotective properties in premenopausal women with T2DM, compared to those in women of the same age without T2DM [23].

The subjects in our study were not taking statin drugs, which reduced the potential confounding impact of this class of medication on lipid profiles and their influence on decreasing SAA-related inflammation [24]. Nevertheless, several participants with T2DM were taking other medications. Metformin, sulphonylurea, DDP-4 inhibitors, GLP1 analogues, and insulin have all been reported to influence lipid levels in a variety of ways [25]. In our study, LDL levels and total cholesterol were comparable between groups, reducing their influence on the outcomes measured. Metformin was the most commonly prescribed medication and previous studies have suggested that metformin does not significantly alter HDL levels nor influence either PON-1 or LCAT activities, minimising the effects of potential confounding [26]. However, metformin has been reported to decrease circulating SAA levels [27]. Exclusion of both insulin-treated patients and/or those taking GLP-1 analogues made no difference to the significance of any of the results reported, with the exception that serum-PON-1 activity was no longer significantly different between T2DM and controls. Reduced sample size may have contributed to the loss of significance detected.

BMI was measured and found to be similar between case and control groups, thus minimising its potential confounding influence, given that increased adiposity and associated obesity are known to be associated with increased inflammation, specifically SAA-related inflammation [28]. As expected, HbA1c levels were higher in the T2DM group and this resulted in a positive correlation between HbA1c and SAA levels, which supports the concept that poor glycaemic control is associated with increased inflammation. Previously, McEneny and colleagues [19] showed that a 1% increase in HbA1c coincided with an increase in HDL_2_-SAA and HDL_3_-SAA of 20% and 23%, respectively, in subjects with type 1 diabetes mellitus (T1DM). Unfortunately, due to limited sample size and power, it was not possible for the current study to investigate this relationship further. However, one could assume that this relationship would extend to subjects with T2DM, although this would require further exploration in a larger T2DM population.

Lower HDL-C levels were reported in subjects with T2DM, while apoAI concentrations were comparable between T2DM and control subjects. This would suggest that the HDL particle size was reduced in subjects with T2DM compared to that in controls, which is suggestive of a proatherogenic phenotype since the HDL particle size has been reported to be inversely associated with CVD risk [29]. Evidence that apoAI is displaced from HDL by SAA [30] would suggest a reduction in the concentration of apoAI in T2DM under elevated SAA levels, although there was little evidence of this in our data. In support of our findings, direct injection of a SAA adenoviral vector into mice or initiating an acute phase response increased SAA's association with HDL but did not result in attenuated apoAI concentration [31, 32]. Both studies suggest that the milieu in which HDL exists provides a dynamic environment where HDL interacts with other lipoproteins and displacement of the apoAI molecule from one HDL particle may simply lead to its reassociation with another in vivo. Under such circumstances, only some of the HDL particles would be apoAI depleted and SAA enriched, with altered particle composition and possibly function, while other HDL particles would be apoAI enriched. Although these studies were conducted in mice, they may provide some rationale as to why increased SAA levels did not result in a net loss of apoAI in our study. McEneny and colleagues [19] also reported an increase in HDL_2_-SAA and HDL_3_-SAA in subjects with T1DM without a change in apoAI concentration. Although their study was conducted in those with T1DM, the mechanistic effect may result from something beyond simple displacement of apoAI by SAA in diabetes. Furthermore, others have reported that apoAI levels alone were insufficient for the prediction of diabetes incidence with the actual apoAI/HDL-C ratio being more informative [33]. Since this study indicates an increase in the ratio of apoAI to HDL-C in subjects with T2DM, it would suggest that these subjects may have more atherogenic HDL particles.

SAA concentrations within serum and HDL_2_ and HDL_3_ subfractions were higher in subjects with T2DM, supporting previously reported findings [34, 35]. When HDL is associated with SAA, it becomes dysfunctional, leading to HDL having increased proatherogenic properties, reduced RCT function, and diminished antioxidant properties, while increasing HDL binding to the arterial wall promoting subsequent atherosclerosis [14–16, 36, 37]. The positive correlations between LDL/VLDL-associated CDs and serum-SAA and HDL_3_-SAA provide some support for increased oxidative stress within LDL/VLDL subfractions where SAA levels are high and which normally HDL would reduce atherogenesis risk [10]. The highly significant correlation (*r* = 0.906; *p* < 0.001) observed between serum-SAA and HDL_3_-SAA adds further support that HDL_3_ is the preferred acceptor of SAA [14], suggesting subsequent dysfunction is likely to occur to a greater extent in this smaller subfraction. McEneny and colleagues investigated the concentration of SAA in individuals of similar age in a cohort of T1DM (nondiabetic controls, mean age 39 years, and T1DM mean age 36 years) [19]. They reported significant increases in HDL_2_-SAA and HDL_3_-SAA_,_ together with a nonsignificant increase in serum-SAA in those with T1DM compared to controls, suggesting a common increase in inflammation in both types of diabetes.

PON-1 is an antioxidant enzyme whose binding to HDL particles is stabilised by apoAI [38]. PON-1 associates with HDL to prevent its oxidation by copper ions as well as reducing the peroxide and aldehyde content of HDL, this interaction also lessens LDL atherogenic potential, thus potentially reducing the risk of development of atherosclerosis [39]. Previous studies have found PON-1 activity to be lower in those with T2DM [40, 41] implicating the impairment of HDL antioxidant properties. However, when those taking insulin and GLP-1 analogues were excluded, serum-PON-1 activity was more comparable between groups, similar to that observed for PON-1 activity in HDL_2_ and HDL_3_, which may result from a lack of change in apoAI concentration that is required for the stabilisation of PON-1 and its association with HDL. Kopprasch and colleagues suggested the duration of T2DM could affect PON-1 activity [42]. Given the mean age of individuals with T2DM in this study was 37 years, which would be considered relatively young for T2DM, the disease duration may not have been sufficient for diabetic complications to develop. This may reflect previous findings that reported comparable PON-1 activity between subjects with T2DM without nephropathy compared to nondiabetic controls, while individuals with T2DM and incipient or overt nephropathy had decreased PON-1 [43]. Recently, the antioxidant role of SAA when combined with HDL has been proposed through the prevention of copper-induced oxidation of lipoproteins in vitro [44] and in serum samples of those with high SAA concentrations [45]. Jayaraman and colleagues suggest that SAA is less effective than displaced apoAI in preventing oxidation, although SAA may partially compensate for its loss [44]. The consensus of previously published literature suggests that SAA imparts a negative effect on the antioxidant properties of HDL, although further analysis would be required to determine the potential antioxidant properties of SAA in studies of sufficient size that also consider the influence of disease duration.

Our study has shown that CETP activity was increased in both HDL_2_ and HDL_3_, similar to that reported by Ståhlman and colleagues [46]. Our data suggests that elevated CETP activity likely reduces HDL-C concentrations and increases small dense HDL particles and subsequent HDL atherogenicity [47, 48], supporting the hypothesis that small HDL particles are a potential mechanistic mediator in T2DM. Increasing CETP activity can result in increased HDL TG and reduced CE content via the exchange of TGs for CEs between HDL and TG rich lipoproteins, altering the stability of the HDL particle, making it more susceptible to rapid clearance from the circulation, resulting in decreased serum HDL-C levels [49]. A strong positive correlation between HDL_3_-SAA and HDL_3_-CETP suggests that higher SAA levels in T2DM lead to increased CETP activity similar to previous reports [47], further supporting the association of HDL_3_ more readily with SAA than HDL_2_. CETP activity was also found to correlate with HbA1c suggesting poor glycaemic control would result in a smaller HDL particle size.

The effects of T2DM on LCAT activity remain controversial [50–52], and the levels reported between groups within our study were comparable. ApoAI is known to activate LCAT and the similar apoAI concentrations observed could account for the lack of difference in the activity of LCAT. Several studies have suggested changes in LCAT activity is related to poor glycaemic control with the increase in the glycation of apoAI and HDL contributing to this change. While the T2DM subjects in our study had higher HbA1c levels compared to controls, the mean HbA1c result was much lower than that reported previously [50], possibly suggesting a lack of change in LCAT activity may be due to better glycaemic control.

## 5. Study Limitations

Several subjects with T2DM were taking medication known to affect lipid levels, which may influence confounding factors, but without further investigation, it cannot be certain if these drugs directly influenced HDL subfraction functionality.

Good glycaemic control is a recommended clinical measure for those with T2DM, as elevated HbA1c leads to increased complications with levels lower than 7% recommended. However, in our study, 33% of those with T2DM had HbA1c levels greater than 7%, which could contribute to potential confounding; however, the small number did not allow us to perform a subanalysis based on HbA1c levels.

The lack of ability to detect any significant difference in PON-1 activity between T2DM and controls in HDL subfractions could be due to several factors, such as limited sample size, the age of onset, and duration of diabetes.

This study has focused only on female participants and as such may not be a reflection of similar observations of a similar male population.

## 6. Conclusions

In conclusion, our data suggests that SAA may be a useful biomarker of inflammation in subjects with T2DM and provides some further mechanistic support to explain why individuals with T2DM have increased risk of atherosclerosis and CVD, partly through the reduced antiatherogenic properties of both HDL_2_ and HDL_3_. Previous studies have tended to focus on vascular and diabetic complications in persons with diabetes >45 years. We studied younger individuals with diabetes (without apparent CVD) to explore if biochemical phenotypes associated with increased risk of CVD and diabetic complications were present at this earlier age.

## Supplementary Material

Supplementary material Appendix 1: Results standardised to apoAI.

## Figures and Tables

**Table 1:** Subject characteristics.

	Control group (*n* = 42)	T2DM group (*n* = 42)	*p*
Age (years)	37.26 (11.77)	37.05 (4.98)	0.914
BMI (kg/m^2^)	33.52 (7.93)	35.88 (7.72)	0.171
Fasting glucose (mmol/L)^∗^	4.94 (4.60, 5.13)	7.78 (6.10, 9.35)	<0.001
HbA1c (%)^∗^	5.42 (5.13, 5.60)	7.26 (6.10, 8.05)	<0.001
Total cholesterol (mmol/L)	4.69 (0.92)	4.50 (0.95)	0.363
Triglycerides (mmol/L)^∗^	1.24 (0.87, 1.70)	1.53 (1.03, 2.10)	0.074
LDL cholesterol (mmol/L)	2.80 (0.77)	2.74 (0.76)	0.730
HDL cholesterol (mmol/L)^∗^	1.20 (1.08, 1.34)	1.03 (0.89, 1.14)	0.004
Metformin (number)	0	26	NA
Sulfonylurea (number)	0	7	NA
DDP-4 inhibitor (number)	0	4	NA
GLP1 analogue (number)	0	8	NA
Insulin (number)	0	5	NA

Results expressed as mean (SD) or as geometric mean (interquartile range) if not normally distributed, where ^∗^ indicates skewed distributions.

**Table 2:** Nonstandardised results.

	Control group (*n* = 42)	T2DM group (*n* = 42)	*p*
Serum			
SAA (mg/L)^∗^	15 (7, 36)	30 (17, 68)	0.002
PON-1 (U/L)	41,109 (5690)	38,245 (7025)	0.043
HDL_2_			
ApoA1 (mg/L)^∗^	167.0 (151.0, 190.9)	162.9 (151.0, 170.3)	0.443
SAA (mg/L)^∗^	0.4 (0.2, 0.7)	1.0 (0.6, 2.2)	<0.001
PON-1 (U/L)^∗^	293.8 (189.4, 471.5)	331.1 (216.2, 466.7)	0.378
CETP (*μ*mol/L)	217.1 (25.1)	232.6 (14.1)	0.001
LCAT (ratio 470/390)	0.974 (0.049)	0.977 (0.046)	0.809
HDL_3_			
ApoA1 (mg/L)	1908 (251.2)	1926 (178.1)	0.704
SAA (*μ*g/L)^∗^	6 (3, 13)	13 (8, 29)	<0.001
PON-1 (U/L)	9656 (4003)	10,551 (2087)	0.203
CETP (*μ*mol/L)	245.2 (41.2)	279.5 (17.7)	<0.001
LCAT (ratio 470/390)	0.945 (0.051)	0.948 (0.047)	0.833

Results expressed as mean (SD) or as geometric mean (interquartile range) if not normally distributed, where ^∗^ indicates skewed distributions.

**Table 3:** Correlations between SAA and subject characteristics (*n* = 84).

	Serum-SAA *r; p*	HDL_2_-SAA *r; p*	HDL_3_-SAA *r; p*
Age	0.011; 0.921	−0.032; 0.775	0.047; 0.669
BMI	0.480; 0.001	0.358; 0.001	0.414; <0.001
Fasting glucose	0.294; 0.009	0.204; 0.076	0.265; 0.021
HbA1c	0.348; 0.002	0.219; 0.056	0.302; 0.008
HDL cholesterol	−0.197; 0.080	0.062; 0.586	−0.141; 0.217
Serum-SAA	—	0.682; <0.001	0.906; <0.001

**Table 4:** Correlations between SAA- and HDL-associated proteins and enzymes (*n* = 84).

	Serum-SAA *r; p*	HDL_2_-SAA *r; p*	HDL_3_-SAA *r; p*
Serum-PON-1	−0.169; 0.125	—	—
HDL_2_-PON-1	—	0.145; 0.191	—
HDL_2_-CETP	—	0.179; 0.104	—
HDL_2_-LCAT	—	0.188; 0.089	—
HDL_3_-PON-1	—	—	0.066; 0.551
HDL_3_-CETP	—	—	0.333; 0.002
HDL_3_-LCAT	—	—	−0.055; 0.623

**Table 5:** Correlations between HbA1c- and HDL-associated proteins and enzymes (*n* = 84).

	HbA1c *r; p*
Serum-PON-1	−0.199, 0.082
HDL_2_-PON-1	−0.074, 0.524
HDL_2_-CETP	0.269, 0.018
HDL_2_-LCAT	0.093, 0.423
HDL_3_-PON-1	0.036, 0.758
HDL_3_-CETP	0.296, 0.009
HDL_3_-LCAT	0.017, 0.883
