# Supplementary material for: Type 2 Diabetes in Young Females Results in Increased Serum Amyloid A and Changes to Features of High Density Lipoproteins in Both HDL2 and HDL3
Source: J Diabetes Res. 2017 May 9;2017:1314864. doi: 10.1155/2017/1314864 (PMC5450179; doi:10.1155/2017/1314864)
Supplement: Supplementary file 1 — Supplementary material Appendix 1: Results standardised to apoAI. [file 1314864.f1.pdf]

Appendix 1 (Supplementary table)

Results standardised to apoAI

	Control group (n=42)	T2DM group (n=42)	t-test
HDL_2			
SAA (µg/L ApoAI) *	2.31 (0.99, 5.10)	5.87 (3.36, 14.37)	<0.001
PON-1 (U/L ApoAI) *	1.76 (1.03, 2.62)	2.03 (1.31, 2.80)	0.280
CETP (µmol/L ApoAI)	1.32 (0.28)	1.43 (0.16)	0.027
LCAT (ratio 470/390 ApoAI)	0.0059 (0.0011)	0.0060 (0.0007)	0.580
HDL_3			
SAA (µg/L ApoAI) *	3.07 (1.56, 7.46)	6.82 (3.86, 14.35)	0.001
PON-1 (U/L ApoAI)	5.25 (2.62)	5.52 (1.19)	0.542
CETP (µmol/L ApoAI)	0.13 (0.03)	0.15 (0.03)	0.006
LCAT (ratio 470/390 ApoAI)	0.0005 (0.0001)	0.0005 (0.0001)	0.535

Results expressed as mean (SD) or as geometric mean (interquartile range) if not normally distributed, where * indicates skewed distributions.
